# Mitochondrial antioxidant elamipretide improves learning and memory impairment induced by chronic sleep deprivation in mice

**DOI:** 10.1002/brb3.3508

**Published:** 2024-04-30

**Authors:** Yue‐Ming Zhang, Ya‐Tao Wang, Ru‐Meng Wei, Xue‐Yan Li, Bao‐Ling Luo, Jing‐Ya Zhang, Kai‐Xuan Zhang, Shi‐Kun Fang, Xue‐Chun Liu, Gui‐Hai Chen

**Affiliations:** ^1^ Department of Neurology (Sleep Disorders) The Affiliated Chaohu Hospital of Anhui Medical University Hefei Anhui P. R. China; ^2^ Department of Neurology The Second People's Hospital of Hefei and Affiliated Hefei Hospital of Anhui Medical University Hefei Anhui P. R. China

**Keywords:** inflammatory, learning and memory, mitochondrial dysfunction, SIRT1, SS‐31, synaptic proteins

## Abstract

**Background:**

The inflammation and synaptic dysfunction induced by mitochondrial dysfunction play essential roles in the learning and memory impairment associated with sleep dysfunction. Elamipretide (SS‐31), a novel mitochondrion‐targeted antioxidant, was proven to improve mitochondrial dysfunction, the inflammatory response, synaptic dysfunction, and cognitive impairment in models of cerebral ischemia, sepsis, and type 2 diabetes. However, the potential for SS‐31 to improve the cognitive impairment induced by chronic sleep deprivation (CSD) and its underlying mechanisms is unknown.

**Methods:**

Adult c57BL/6J mice were subjected to CSD for 21 days using an activity wheel accompanied by daily intraperitoneal injection of SS‐31 (5 mg/kg). The novel object recognition and Morris water maze test were used to evaluate hippocampus‐dependent cognitive function. Western blotting and reverse transcription‐quantitative polymerase chain reaction assays were used to determine the effects of CSD and SS‐31 on markers of mitochondria, inflammation response, and synaptic function. Enzyme‐linked immunosorbent assays were used to examine the levels of proinflammatory cytokines.

**Results:**

SS‐31 could improve the cognitive impairment induced by CSD. In particular, SS‐31 treatment restored the CSD‐induced decrease in sirtuin 1 (SIRT1) and peroxisome proliferator‐activated receptor γ coactivator alpha levels and the increase in levels nuclear factor kappa‐B and inflammatory cytokines, including interleukin (IL)‐1β, IL‐6, and tumor necrosis factor‐alpha. Furthermore, SS‐31 significantly increased the levels of brain‐derived neurotrophic factor, postsynaptic density protein‐95, and synaptophysin in CSD mice.

**Conclusion:**

Taken together, these results suggest that SS‐31 could improve CSD‐induced mitochondrial biogenesis dysfunction, inflammatory response, synaptic dysfunction, and cognitive impairment by increasing SIRT1 expression levels.

## 1 INTRODUCTION

Sleep is a conserved biological behavior in mammals. Classical theories posit that sleep is driven by two major factors: (I) a homeostatic mechanism controlled by sleep‐promoting substances, including adenosine and prostaglandin E; and (II) a circadian clock that is mainly regulated by the suprachiasmatic nucleus and other rhythmic nuclei (Chung et al., 2017; Huang et al., 2014; Weber & Dan, 2016). The daily variations during rest and activity periods are regulated by homeostasis and rhythm but are highly susceptible to external interference. Accumulating evidence indicates that adequate sleep is conducive to removing brain waste products, energy recovery, and memory consolidation (Stickgold & Walker, 2005; Xie et al., 2013). With the development of society, the prevalence of sleep dysfunction has increased in many populations, mostly due to coffee abuse, electronic devices use, and heavy workloads, which in turn increases the risk of neurological and psychotic disorders, including anxiety, depression, and cognitive impairment (Cai et al., 2022; Xie et al., 2020; Yang et al., 2023).

Given the well‐known cognitive effects and increasing prevalence of sleep dysfunction in society, investigating the mechanism underlying sleep deprivation (SD)‐induced cognitive deficits has become a research hotspot. Previous studies on the mechanisms underlying the cognitive impairment associated with SD largely focused on changes in synaptic proteins, synaptic plasticity, the inflammatory response, and neurotransmitters. For example, acute SD for 48 h induced by an automated cage‐shaking stimulus resulted in location recognition memory impairment and reduced protein expression levels of synaptophysin (SYN), synapsin, and postsynaptic density‐95 (PSD‐95) in the hippocampus of rats (Wadhwa et al., 2015). Nuclear factor kappa‐B (NF‐κB) is a classic inflammatory regulator and its activation leads to the release of pro‐inflammatory cytokines, including interleukin (IL)‐1β, IL‐6, and tumor necrosis factor‐alpha (TNF‐α). Chronically repeated paradoxical SD for 3 months significantly increased NF‐κB activity and the level of TNF‐α in the hippocampus, leading to anxiety and working memory dysfunction in mice (Yin et al., 2017). However, research in this field to date has neglected the influences of SD on the intrinsic properties of neurons.

Growing evidence suggests that mitochondrial dysfunction is involved in the cognitive dysfunction induced by SD. In addition to their primary function as intracellular organelles within neurons responsible for producing adenosine triphosphate (ATP), the mitochondria also perform other essential cellular functions, including regulating the inflammatory response, synaptic plasticity, energy metabolism, production and elimination of reactive oxygen species (ROS), and cell survival and death (Ahmad et al., 2023; Chen et al., 2023). SD was reported to impair the mitochondrial electron transport system, accompanied by an increase in ROS production and lipid peroxidation (Rodrigues et al., 2018). Paradoxical SD decreased the membrane excitability of CA1 pyramidal neurons by translocating Bax to the mitochondria and releasing cytochrome C into the cytoplasm (Yang et al., 2008). In view of the above evidence, it appears possible that the inflammation, synaptic dysfunction, and cognitive impairment induced by SD could be improved by pharmacological methods targeting damaged mitochondria.

Elamipretide (SS‐31; d‐Arg‐dimetthyl‐Tyr‐Lys‐Phe‐NH2) is a mitochondrion‐targeted antioxidant discovered by Hazel Szeto and Peter Schiller. Owing to its aromatic cationic structure, SS‐31 can freely cross the blood–brain barrier and cell membrane, ultimately reaching a concentration of more than 1000 times in the mitochondria independent of mitochondrial membrane potential (Petri et al., 2006; Szeto, 2008; Siegel et al., 2013). SS‐31 has been found to scavenge various mitochondrial ROS, improve ATP production, prevent mitochondrial swelling, and decrease the inflammatory response by binding to mitochondrial cardiolipin (Birk et al., 2013; Hao et al., 2015). Furthermore, SS‐31 was reported to activate the downstream signaling pathways related to peroxisome proliferator‐activated receptor γ co‐activator alpha (PGC‐1α), a master regulator of mitochondrial activity that is involved in regulating mitochondrial biogenesis and function, by modulating sirtuin 1 (SIRT1) levels (Escribano‐Lopez et al., 2018). SS‐31 has already been shown to act as a protective factor in models of ischemia, sepsis, and traumatic brain injury by reversing mitochondrial function (Cai et al., 2018; Wu, Zhang, et al., 2015; Zhu et al., 2018). However, the potential protective effects of SS‐31 on chronic sleep deprivation (CSD)‐induced learning and memory impairment and underlying mechanisms remain unclear.

We hypothesized that SS‐31 would improve the inflammatory response and synaptic dysfunction induced by CSD by repairing mitochondrial function to ultimately alleviate the cognitive deficits. To test this hypothesis, we created a mouse model of CSD and examined the effects of SS‐31 administration on learning and memory function, markers of mitochondrial and synaptic function, and proinflammatory cytokines.

## 2 MATERIALS AND METHODS

### 2.1 Animals

Male 2‐month‐old C57BL/6J mice were obtained from Beijing Vital River Laboratory Animal Technology Co., Ltd. (specific pathogen‐free grade). The animals are kept in an environment where food and water are available ad libitum, with a 12‐h dark–light cycle, 21–23°C temperature and 50%–60% relative humidity.

### 2.2 Experimental protocols

**FIGURE 1:**
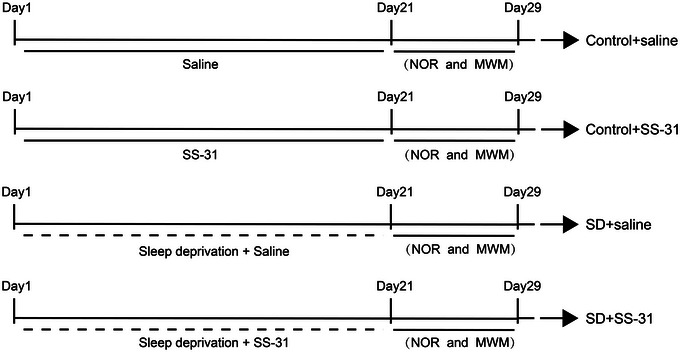
Experimental protocol. MWM, Morris water maze test; NOR, novel object recognition; SD, sleep deprivation.

The male C57BL/6J mice were randomly allocated to one of the following four groups (*n* = 8 per group): Control + saline, Control + SS‐31, SD + saline, and SD + SS‐31. SS‐31 or saline (5 mg/kg) was administered intraperitoneally to the mice 30 min prior to sleep deprivation using an adapted version of the BW‐NSD404 sleep deprivation machine (Shanghai Bio‐will Co., Ltd.). The principle of the machine is to place the mouse on a platform that is constantly moving to prevent the mouse from falling asleep (Zhang, Cheng, et al., 2022). After administration of SS‐31 or saline, the mice in the SD + SS‐31 and SD + saline groups were subjected to the sleep deprivation machine for 21 consecutive days from 2:00 p.m. to the following day at 8:00 a.m. (Konakanchi et al., 2023), whereas mice in the Control + saline and Control + SS‐31 groups were maintained on the static (not moving) machine during this same period (see Figure 1).

### 2.3 Novel object recognition test

The new object experiment was used to assess short‐term cognitive function in mice. As previously performed by others (Leger et al., 2013), the experiment was divided into three parts: habituation, familiarization, and test phases. In the habituation phase, mice were placed in an empty open field and explored freely for 5 min. A period of 24 h later, mice were placed in an open field containing two identical objects for familiarization, and the experiment was discontinued when both objects had been explored for 20 s or when the 10‐min had expired. During the test phase, one of the objects in the open field was replaced with a new object and the mice were allowed to explore the old (D1) and new objects (D2) freely with 10 min. A 75% alcohol was used at the end of each experiment to eliminate the effect of odor. The memorization ability was quantified by novel object recognition index (NOI): NOI = D2/(D1 + D2).

### 2.4 Morris water maze test

To avoid the influence of the novel object recognition test on the subsequent Morris water maze test, the Morris water maze test was conducted on a new cohort of the mice. The protocol used in this study was similar to the one previously described and was used to assess the spatial learning and memory abilities of mice (Zhang, Cheng, et al., 2022). The test was divided into a learning phase and a memory phase. During the learning phase, mice were trained in a circular white pool with a diameter of 120 cm and a height of 30 cm. Each mouse performed four trials per day to find the hidden target platform and was allowed to rest on the platform for 30 s whether or not they found the hidden platform within 60 s. The learning phase lasted for a total of 7 days. The target underwater platform was removed 2 h after the last experiment on the last day of the learning phase. During the memory phase, after the hidden platform was removed, the mice entered the water from the opposite quadrant of the quadrant where the target platform was located and explored freely for 60 s. ANY‐maze tracking system was used for recording and analysis of all trials.

### 2.5 Reverse transcription‐fluorescence quantitative polymerase chain reaction (RT‐PCR)

Total RNA was extracted from the hippocampal tissue by adding Trizol lysate. Spectrophotometer was used to assess the purity of the extracted RNA. The RNA was reverse‐transcribed to cDNA using the PrimeScript reverse transcription (RT) reagent Kit with gDNA Eraser (TaKaRa, RR047A). The transcripts were then amplified by quantitative polymerase chain reaction (qPCR). The cDNA was used as a template and the reaction system was as follows: 5 µL of 2× SYBR Green Mixture, 1 µL of forward primer, 1 µL of reverse primer, 2 µL of RNase‐free water, and 1 µL of cDNA. The reaction conditions were a single cycle of pre‐denaturation at 95°C for 1 min, followed by a total of 40 cycles at 95°C for 20 s and 60°C for 1 min. The primers are designed by Primer 3 software and synthesized by Sangon Biotech company. The amplification profile of PCR showed *S* curve and the level of mRNA was quantified using the 2^−ΔΔ^ *^Ct^* method. Table 1 presents the primer sequences.

**TABLE 1:** Primer sequences.

Gene	Amplicon size (bp)	Forward primer (5′ → 3′)	Reverse primer (5′ → 3′)
β‐actin	120	AGTGTGACGTTGACATCCGT	TGCTAGGAGCCAGAGCAGTA
Sirt1	116	TAATGTGAGGAGTCAGCACC	GCCTGTTTGGACATTACCAC
Pgc1α	114	TGTGACTGGGGACTGTAGTA	AGAGCAGCACACTCTATGTC
Nfκb	119	GCTCCTGTTCGAGTCTCCAT	TTGCGCTTCTCTTCAATCCG
Bdnf	94	TTACTCTCCTGGGTTCCTGA	ACGTCCACTTCTGTTTCCTT
Trkb	104	TCTGGAGGGTGCTATGCTAT	GGGGCAGAAACTCCAGAAAA
Psd95	72	CCCAGGATATGTGAACGGAA	CCTGAGTTACCCCTTTCCAA
Syn	124	GCCTACCTTCTCCACCCTTT	GCACTACCAACGTCACAGAC

Abbreviations: Bdnf, brain‐derived neurotrophic factor; Pgc1α, proliferator‐activated receptor γ co‐activator alpha; Psd95, postsynaptic density‐95; Sirt1, sirtuin 1; Syn, synaptophysin.

### 2.6 Western blotting (WB)

The protocol of western blotting (WB) was performed as previously described (Wei et al., 2022). In brief, the hippocampal tissue was lysed in RIPA lysis buffer (Beyotime, P0013B) and centrifuged at 12,000 × *g* for 15 min to collect the supernatant. The protein samples were electrophoretically separated and then blotted onto polyvinylidene fluoride membranes (Millipore, IPVH00010). The protein levels were determined via incubation with antibodies against PGC‐1α (1:1000; abcam), SIRT1 (1:500; Santa Cruz), NF‐κB (1:5000; abcam), brain‐derived neurotrophic factor (BDNF; 1:1000; abcam), PSD‐95 (1:2000; abcam), SYN (1:1000; bioss), IL‐1β (1:500; bioss), IL‐6 (1:1000; Wanleibio), and TNF‐α (1:1000; bioss). The membranes were then incubated with horseradish peroxidase‐labeled secondary antibodies (1:20,000; Zsbio) according to the properties of the primary antibody. Protein detection was performed using an ECL luminescence kit (Thermo, 340958) and quantified with ImageJ software.

### 2.7 Enzyme‐linked immunosorbent assay (ELISA)

The concentrations of inflammatory factors (IL‐1β, IL‐6, and TNF‐α) were detected in the hippocampal samples using corresponding enzyme‐linked immunosorbent assay (ELISA) kits from Wuhan ColorfulGene Biological Technology Co., Ltd. (JYM0531Mo, JYM0012Mo, and JYM0218Mo, respectively). All experiments were performed in accordance with the manufacturer's instructions.

### 2.8 Data analysis

All data are normal and expressed as mean ± standard errors of the mean. Repeated‐measures analysis of variance was used to analyze the data from the learning period of the Morris water maze test, whereas two‐way analysis of variance was used to analyze the data from the memory period of the Morris water maze test, novel object recognition, WB, RT‐qPCR, and ELISA with the Tukey post hoc test to compare the differences among the four groups. The correlations between variables were determined using Pearson's correlation coefficient. All data analyses were performed using GraphPad 8.0 software. *p* < .05 was considered statistically different.

## 3 RESULTS

### 3.1 SS‐31 improved CSD‐induced recognition memory deficits

**FIGURE 2:**
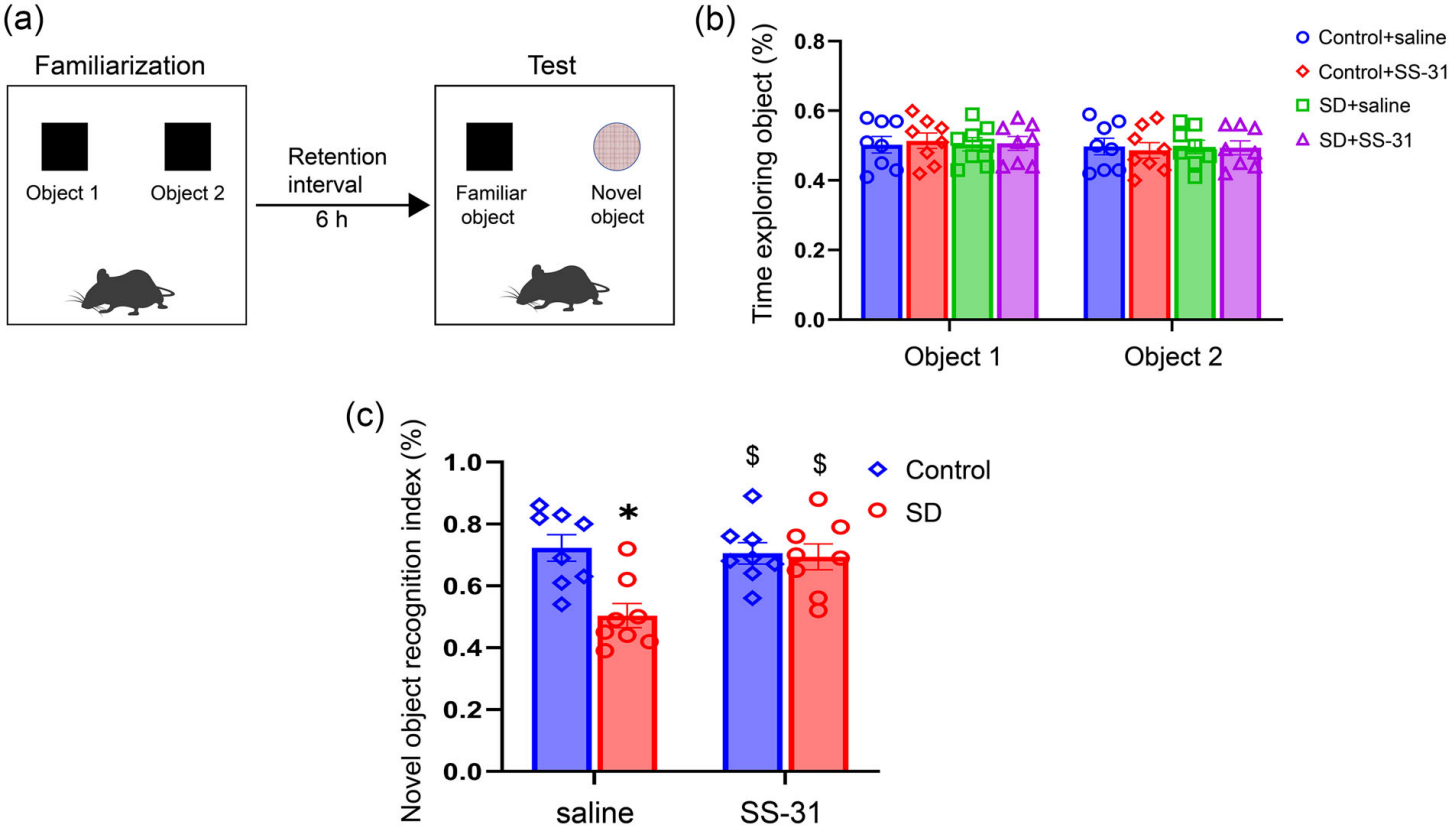
SS‐31 improved recognition memory in the novel object recognition test after sleep deprivation (SD) in mice: (a) Schematic of the novel object recognition test; (b) the percent time of each experimental group mice exploring two objects (object 1 and object 2) during familiarization phase; (c) the novel object recognition index of each experimental group during test phase. Two‐way ANOVA and Tukey's post hoc test, ^*^ *p* < .05 in comparison with the Control + saline group; ^$^ *p* < .05 in comparison with the SD + saline group.

First, the novel object recognition test was used to evaluate whether SS‐31 could improve recognition memory deficits induced by CSD. During the familiarization phase, there was no significant difference in the exploration time for two same objects among the four groups (*p* > .05, Figure 2a,b). In the test phase, the NOI of SD + saline group was significantly lower than that in Control + saline group (treatment: *F*_(1, 28)_ = 8.37, *p* < .01; drug: *F*_(1, 28)_ = 4.71, *p* < .05; treatment × drug: *F*_(1, 28)_ = 6.81, *p* < .05, Figure 2C). Furthermore, SS‐31 treatment reversed this decline in the SD + SS‐31 when compared to SD + saline group (*p* < .05). These results suggested that SS‐31 treatment could ameliorate the recognition memory impairment induced by CSD.

### 3.2 SS‐31 improved CSD‐induced spatial learning and memory impairment

**FIGURE 3:**
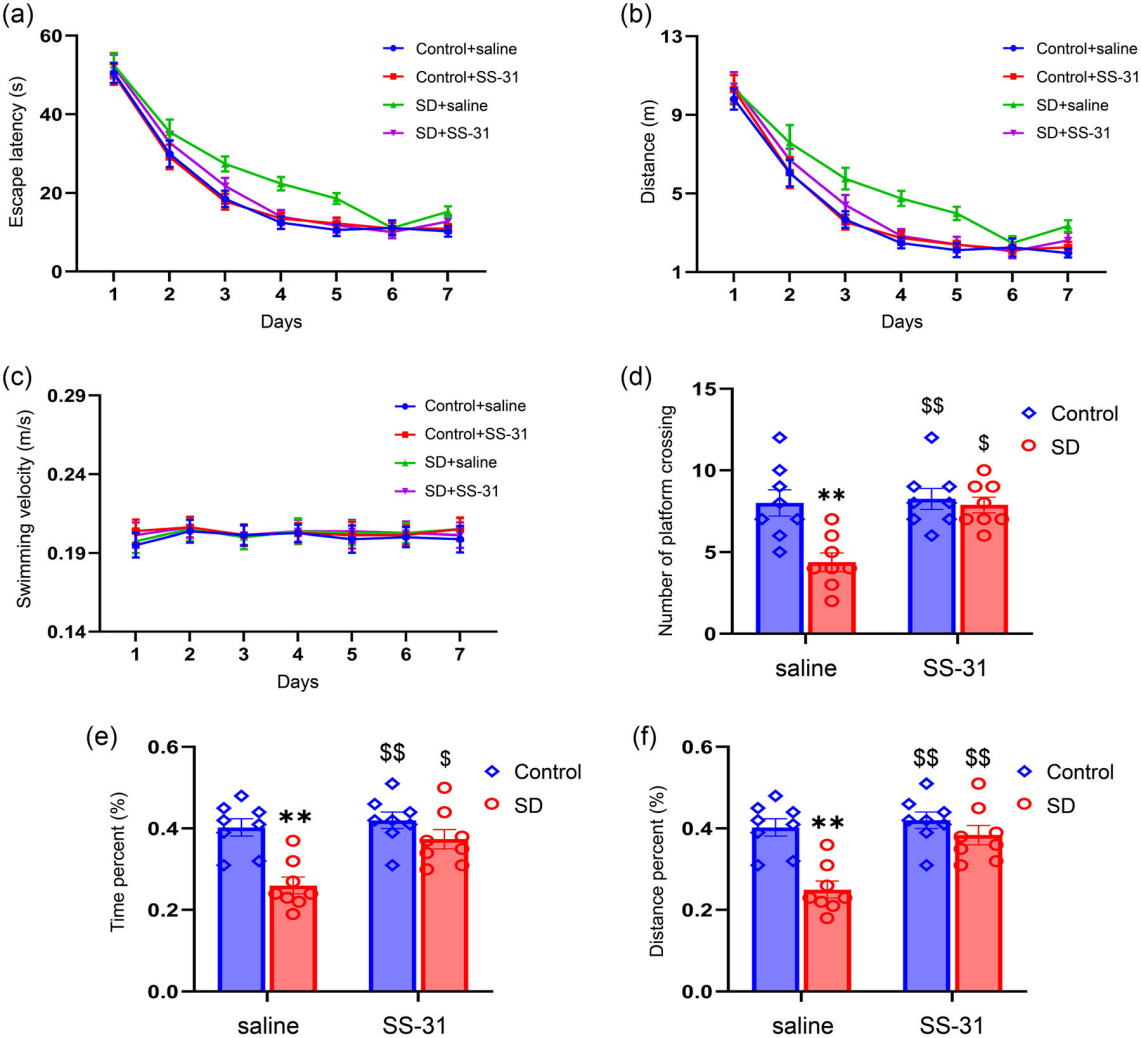
SS‐31 improved behavioral performance in the Morris water maze test after sleep deprivation (SD) in mice: (a and b) Each experimental group of mice learned to locate the hidden platform at the learning phase; (c) comparison of swimming velocity among the four groups; (d) the number of platform‐crossing in the memory phase of the Morris water maze test; (e and f) comparison of percent time and distance among the experimental groups during the memory phase. Two‐way ANOVA and Tukey's post hoc test, ^**^ *p* < .01 in comparison with the Control + saline group; ^$^ *p* < .05 in comparison with the SD + saline group, ^$$^ *p* < .01 in comparison with the SD + saline group.

The Morris water maze test was used to assess the influence of CSD and SS‐31 on hippocampal‐dependent learning and memory. On the first day of the learning phase, there was no difference in the time of searching hidden platform in the four quadrants, indicating that the mice from four groups did not show a preference for any quadrant at the beginning of the learning phase (supplementary file Table 1). The escape latency and distance gradually decreased with an increase in the training days (escape latency: *F*_(6,168)_ = 207.47, *p* < .01; distance: *F*_(6,168)_ = 158.24, *p* < .01; Figure 3a,b). With respect to treatment effects, there were significant differences in escape latency and distance among the four groups (escape latency: *F*_(3,28) _= 10.10, *p* < .01; distance: *F*_(3,28)_ = 8.93, *p* < .01; Figure 3A,B). The post hoc analysis documented that the mice from the SD + saline group spent a longer time to find the hidden platform than the mice from the Control + saline and Control + SS‐31 groups (*Ps* < .1). There was also a significant difference between the SD + saline and SD + SS‐31 groups (*p* < .01). No differences were observed in swimming velocity among the different treatment groups (Figure 3C).

In the memory phase, the number of platform‐crossing, percent time, and distance showed significant differences among the four groups (number of platform‐crossing: treatment: *F*_(1, 28)_ = 9.93, *p* < .01; drug: *F*_(1, 28)_ = 8.73, *p* < .01; treatment × drug: *F*_(1, 28)_ = 6.55, *p* < .05; time: treatment: *F*_(1, 28)_ = 19.12, *p* < .01; drug: *F*_(1, 28)_ = 9.25, *p* < .01; treatment × drug: *F*_(1, 28)_ = 4.97, *p* < .05; distance: treatment: *F*_(1, 28)_ = 19.12, *p* < .01; drug: *F*_(1, 28)_ = 12.28, *p* < .01; treatment × drug: *F*_(1, 28)_ = 7.25, *p* < .05; Figure 3d–f, supplementary file Table 2). The post hoc analyses showed that the number of platform‐crossing, percent time, and distance were shorter in the SD + saline group than in the Control + saline group (*p* < .05). There was also a significant difference between the SD + saline and SD + SS‐31 groups (*p* < .05).

### 3.3 SS‐31 reverses the CSD‐induced changes to SIRT1, PGC‐1α, and NF‐κB levels

**FIGURE 4:**
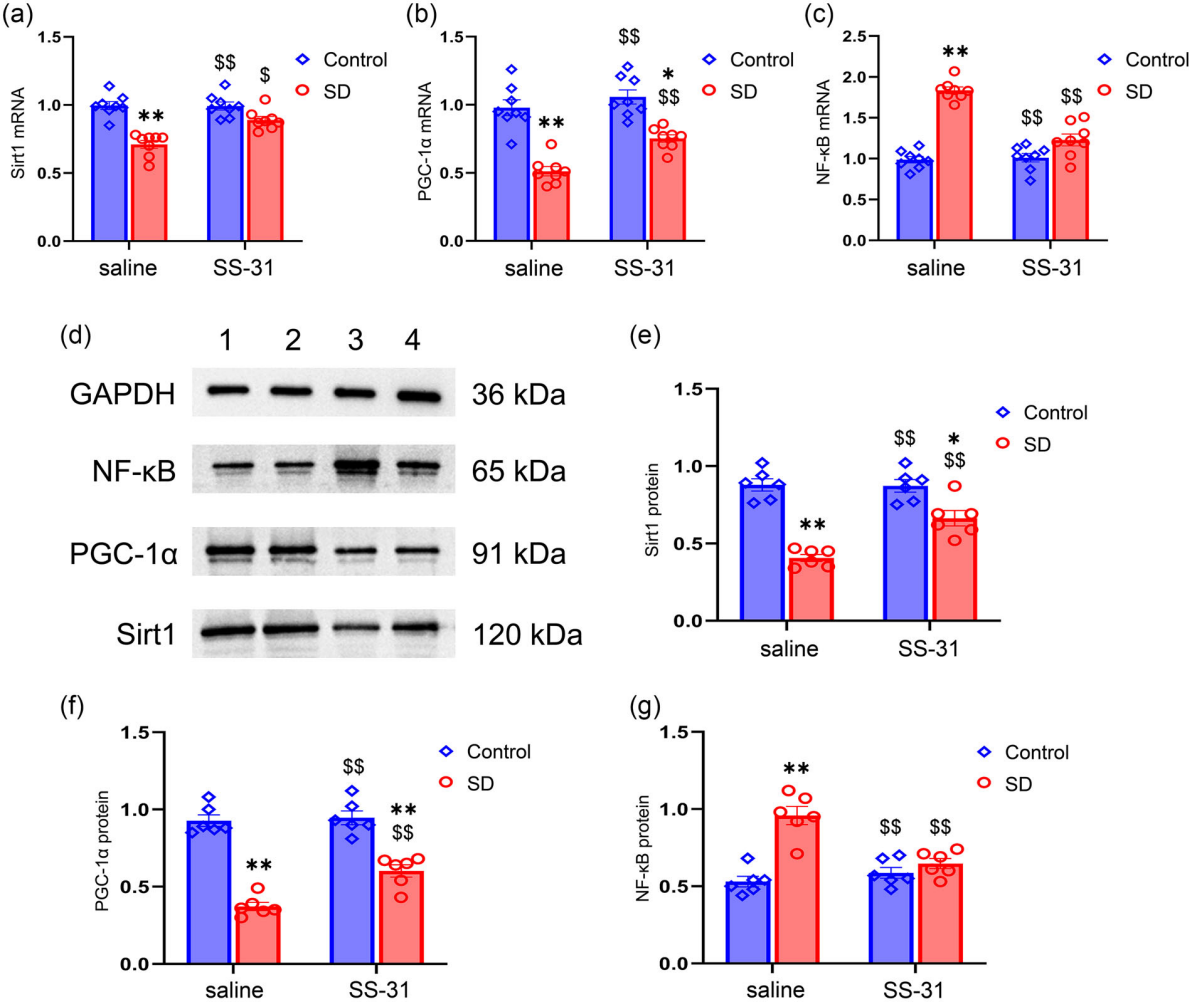
Effects of sleep deprivation (SD) and SS‐31 on the mRNA and protein levels of sirtuin 1 (SIRT1), peroxisome proliferator‐activated receptor γ coactivator alpha (PGC‐1α), and nuclear factor kappa‐B (NF‐κB): (a–c) Quantification of mRNA levels among the four groups; (d) representative SIRT1, PGC‐1α, and NF‐κB immunoreactive bands in the hippocampus of each experimental group: band 1, Control + saline; band 2, Control + SS‐31; band 3, SD + saline; band 4, SD + SS‐31; (e–g) quantification of protein levels among the four groups. Two‐way ANOVA and Tukey's post hoc test, ^*^ *p* < .05 in comparison with the Control + saline group, ^**^ *p* < .01 in comparison with the Control + saline group; ^$^ *p* < .05 in comparison with the SD + saline group, ^$$^ *p* < .01 in comparison with the SD + saline group.

The mRNA levels of *Sirt1*, *Pgc1a*, and *Nfkb* were different among the four groups (*Sirt1*: treatment: *F*_(1, 28)_ = 46.93, *p* < .01; drug: *F*_(1, 28)_ = 9.65, *p* < .01; treatment × drug: *F*_(1, 28)_ = 10.48, *p* < .01; *Pgc1a*: treatment: *F*_(1, 28)_ = 75.10, *p* < .01; drug: *F*_(1, 28)_ = 12.83, *p* < .01; treatment × drug: *F*_(1, 28)_ = 3.28, *p* > .05; *Nfkb*: treatment: *F*_(1, 28)_ = 101.40, *p* < .01; drug: *F*_(1, 28)_ = 30.18, *p* < .01; treatment × drug: *F*_(1, 28)_ = 35.59, *p* < .01; Figure 4A–C, Figure S1). Post hoc analysis revealed that the mRNA expression of *Sirt1* and *Pgc1a* was downregulated, whereas the expression of *Nfkb* mRNA was upregulated after CSD. Furthermore, SS‐31 treatment counteracted the effects induced by CSD.

Similarly, the protein levels of SIRT1, PGC‐1α, and NF‐κB were different among the four groups (SIRT1: treatment: *F*_(1, 20)_ = 74.25, *p* < .01; drug: *F*_(1, 20)_ = 10.04, *p* < .01; treatment × drug: *F*_(1, 20)_ = 11.13, *p* < .01; PGC‐1α: treatment: *F*_(1, 20)_ = 144.20, *p* < .01; drug: *F*_(1, 20)_ = 10.94, *p* < .01; treatment × drug: *F*_(1, 20)_ = 7.95, *p* < .05; NF‐κB: treatment: *F*_(1, 20)_ = 34.91, *p* < .01; drug: *F*_(1, 20)_ = 9.52, *p* < .01; treatment × drug: *F*_(1, 20)_ = 19.86, *p* < .01; Figure 4d–g, Figure S1). Post hoc analysis showed that the protein levels of SIRT1 and PGC‐1α were decreased after CSD, which were restored with SS‐31 treatment. The protein level of NF‐κB was increased in the SD + saline group when compared to that of the Control + saline group, and this effect was also reversed by SS‐31 treatment.

### 3.4 SS‐31 suppresses the CSD‐induced increase in proinflammatory cytokines

**FIGURE 5:**
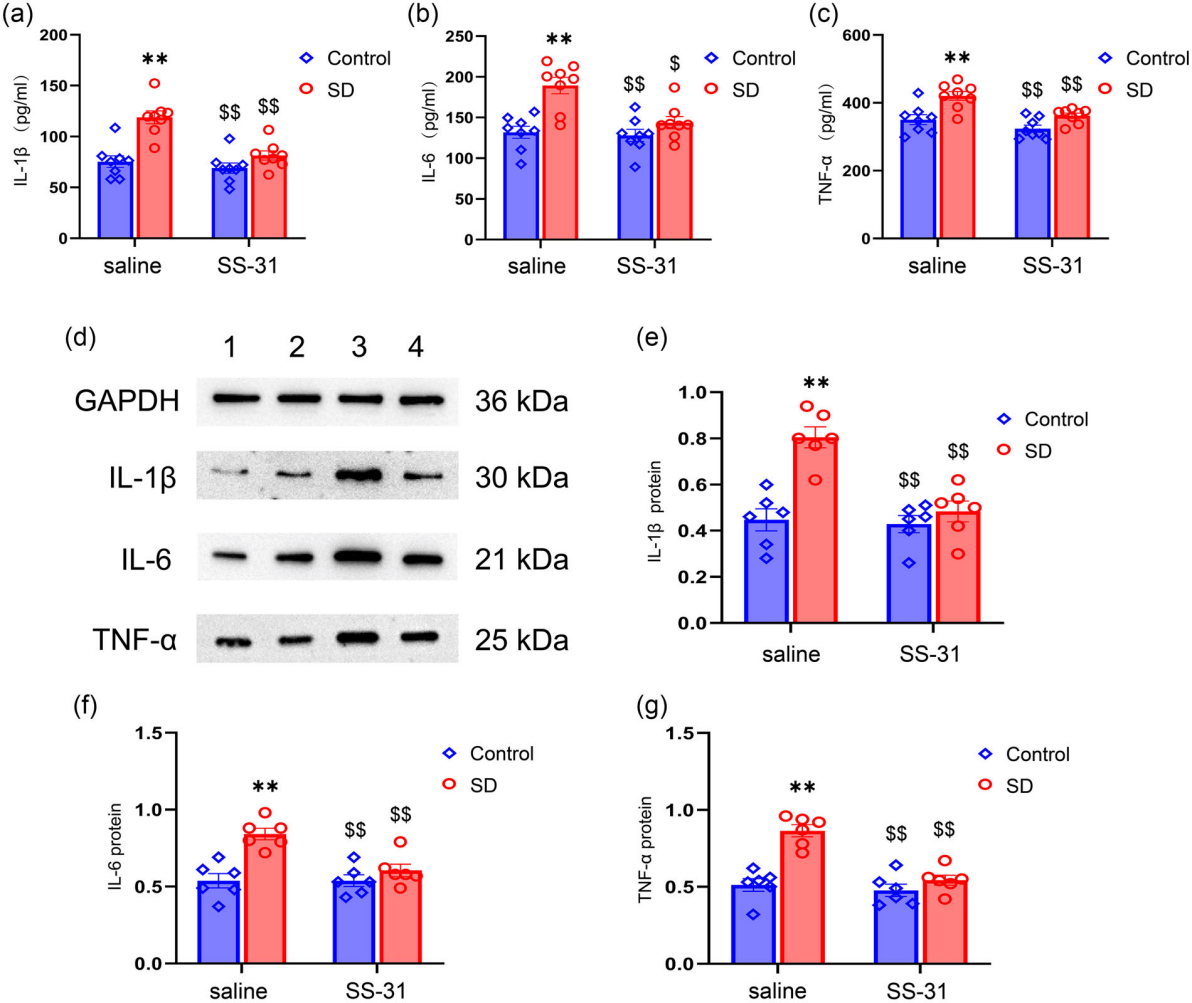
Effects of sleep deprivation (SD) and SS‐31 on the expression levels of the proinflammatory cytokines: (a–c) Quantification of interleukin (IL)‐1β, IL‐6, and tumor necrosis factor‐alpha (TNF‐α) levels by enzyme‐linked immunosorbent assay; (d) representative IL‐1β, IL‐6, and TNF‐α immunoreactive bands in the hippocampus of each experimental group: band 1, Control + saline; band 2, Control + SS‐31; band 3, SD + saline; band 4, SD + SS‐31; (e–g) quantification of IL‐1β, IL‐6, and TNF‐α levels by western blotting. Two‐way ANOVA and Tukey's post hoc test, ^**^ *p *< .01 in comparison with the Control + saline group; ^$^ *p* < .05 in comparison with the SD + saline group, ^$$^ *p* < .01 in comparison with the SD + saline group.

The expression levels of IL‐1β, IL‐6, and TNF‐α were significantly different among the four groups (**ELISA**: IL‐1β: treatment: *F*_(1, 28)_ = 26.30, *p* < .01; drug: *F*_(1, 28)_ = 16.17, *p* < .01; treatment × drug: *F*_(1, 28)_ = 8.25, *p* < .01; IL‐6: treatment: *F*_(1, 28)_ = 19.71, *p* < .01; drug: *F*_(1, 28)_ = 9.09, *p* < .01; treatment × drug: *F*_(1, 28)_ = 6.49, *p* < .05; TNF‐α: *F*_(1, 28)_ = 21.26, *p* < .01; drug: *F*_(1, 28)_ = 13.15, *p* < .01; treatment × drug: *F*_(1, 28)_ = 1.82, *p* > .05; Figure 5a–c **; WB**: IL‐1β: treatment: *F*_(1, 20)_ = 21.88, *p* < .01; drug: *F*_(1, 20)_ = 14.80, *p* < .01; treatment × drug: *F*_(1, 20)_ = 11.78, *p* < .01; IL‐6: treatment: *F*_(1, 20)_ = 20.54, *p* < .01; drug: *F*_(1, 20)_ = 8.40, *p* < .01; treatment × drug: *F*_(1, 20)_ = 8.40, *p* < .01; TNF‐α: *F*_(1, 20)_ = 29.58, *p* < .01; drug: *F*_(1, 20)_ = 21.33, *p* < .01; treatment × drug: *F*_(1, 28)_ = 13.78, *p* < .01; Figure 5d–g, Figure S2). Post hoc analyses showed upregulation expression of IL‐1β, IL‐6, and TNF‐α in the SD + saline group when compared to the Control + saline group; however, SS‐31 immediately improved the increase in the levels of IL‐1β, IL‐6, and TNF‐α.

### 3.5 SS‐31 restores the CSD‐induced changes in synaptic plasticity‐associated proteins

**FIGURE 6:**
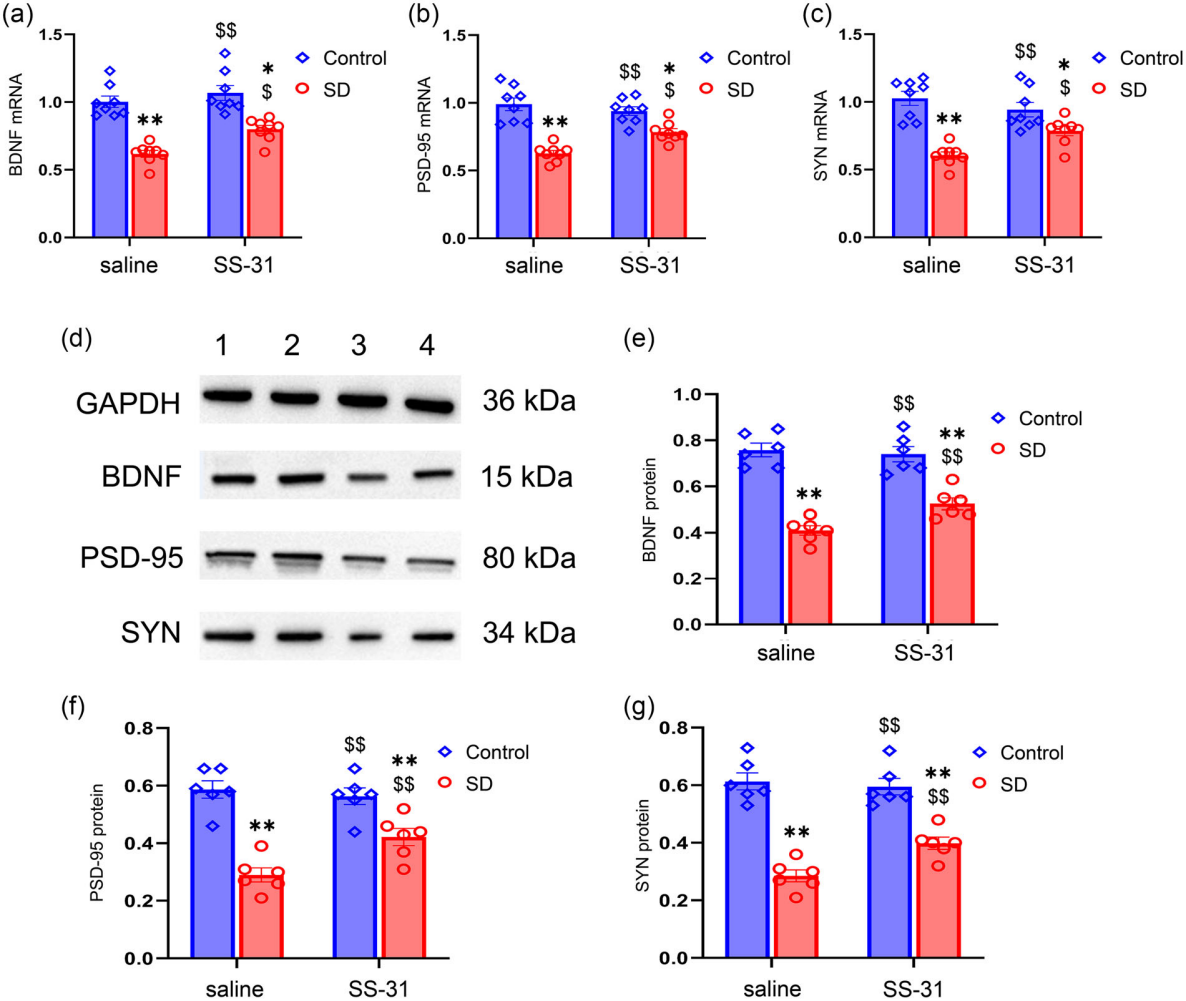
Effects of sleep deprivation (SD) and SS‐31 on the mRNA and protein levels of synaptic plasticity‐associated proteins: (a–c) Quantification of the mRNA levels of brain‐derived neurotrophic factor (BDNF), postsynaptic density‐95 (PSD‐95), and synaptophysin (SYN) among the four groups; (d) representative BDNF, PSD‐95, and SYN immunoreactive bands in the hippocampus of each experimental group: band 1, Control + saline; band 2, Control + SS‐31; band 3, SD + saline; band 4, SD + SS‐31; (e–g) quantification of the protein levels of BDNF, PSD‐95, and SYN among the four groups. Two‐way ANOVA and Tukey's post hoc test, ^*^ *p* < .05 in comparison with the Control + saline group, ^**^ *p* < .01 in comparison with the Control + saline group; ^$^ *p* < .05 in comparison with the SD + saline group, ^$$^ *p* < .01 in comparison with SD + saline group.

The mRNA levels of *Bdnf*, Psd95, and *Syn* were significantly different among the four groups (*Bdnf*: treatment: *F*_(1, 28)_ = 68.71, *p* < .01; drug: *F*_(1, 28)_ = 9.75, *p* < .01; treatment × drug: *F*_(1, 28)_ = 2.17, *p* > .05; *Psd95*: treatment: *F*_(1, 28)_ = 62.22, *p* < .01; drug: *F*_(1, 28)_ = 2.66, *p* < .01; treatment × drug: *F*_(1, 28)_ = 9.91, *p* < .01; *Syn*: treatment: *F*_(1, 28)_ = 45.42, *p* < .01; drug: *F*_(1, 28)_ = 1.28, *p* > .05; treatment × drug: *F*_(1, 28)_ = 9.48, *p* < .01; Figure 6a–c). The post hoc analyses revealed that the mRNA levels of all three of these synaptic plasticity‐associated factors were decreased in the SD + saline group compared with those of the Control + saline and Control + SS‐31 groups (*Ps* < .01). SS‐31 treatment attenuated the decrease of Bdnf, Psd‐95, and Syn mRNA levels in the SD + SS‐31 group (*Ps* < .05).

The protein expression level of BDNF was also significantly different among the four groups (treatment: *F*_(1, 20)_ = 104.40, *p* < .01; drug: *F*_(1, 20)_ = 3.07, *p* > .05; treatment × drug: *F*_(1, 20)_ = 5.85, *p* < .05; Figure 6d,e **;** Figure S3). Post hoc analysis showed that the expression level of BDNF was decreased in the SD + saline group compared with that in the Control + saline group (*p* < .01), whereas SS‐31 treatment restored the BDNF protein content (*p* < .01). The protein expression levels of PSD‐95 and SYN were different among the four groups (PSD‐95: treatment: *F*_(1, 20)_ = 59.09, *p* < .01; drug: *F*_(1, 20)_ = 3.61, *p* > .05; treatment × drug: *F*_(1, 20)_ = 7.39, *p* < .05; SYN: treatment: *F*_(1, 20)_ = 106.90, *p* < .01; drug: *F*_(1, 20)_ = 3.50, *p* > .05; treatment × drug: *F*_(1, 20)_ = 6.73, *p* < .05; Figure 6f,g; Figure S3). The post hoc analysis showed that the protein expression of PSD‐95 and SYN was downregulated in the SD + saline group compared to that of the Control + saline group (*Ps* < .01), whereas SS‐31 significantly attenuated the decrease of PSD‐95 and SYN protein levels in the Control + SS‐31 group (*Ps* < .05).

### 3.6 Morris water maze performance indices are correlated with markers of mitochondrial biogenesis, inflammation, and synaptic function

The escape latency and distance were negatively correlated with the levels of SIRT1, PGC‐1α, BDNF, PSD‐95, SYN, and were positively correlated with the levels of NF‐κB and pro‐inflammatory cytokines including IL‐1β, IL‐6, and TNF‐α in the learning phase. In the memory phase, the percent time and distance were positively correlated with the levels of SIRT1, PGC‐1α, BDNF, PSD‐95, and SYN and were negatively correlated with the levels of NF‐κB and pro‐inflammatory cytokines, as shown in Tables 2, 3, 4.

**TABLE 2:** Correlation between the performance of the Morris water maze test (MWM) and hippocampal levels of IL‐1β, IL‐6, and tumor necrosis factor‐alpha (TNF‐α) [*r* (*p*)].

Tasks	Indexes	Groups	Proinflammatory cytokines		
			IL‐1β	IL‐6	TNF‐α
Morris water maze test	Escape latency	Control + saline	0.801 (0.017)*	−0.190 (0.653)**	0.041 (0.924)
		Control + SS‐31	0.776 (0.024)*	0.752 (0.031)*	0.898 (0.002)**
		SD + saline SD + SS‐31	0.791 (0.020)* 0.853 (0.007)**	0.690 (0.058) 0.842 (0.009)**	0.822 (0.012)* 0.799 (0.017)*
	Distance	Control + saline Control + SS‐31	0.834 (0.010)* 0.823 (0.012)*	−0.102 (0.810) 0.769 (0.026)*	0.243 (0.562) 0.802 (0.017)*
		SD + saline	0.866 (0.005)**	0.807 (0.015)*	0.885 (0.003)**
		SD + SS‐31	0.709 (0.049)*	0.763 (0.028)*	0.783 (0.022)*
	Percentage of distance swam	Control + saline	−0.843 (0.009)**	−0.117 (0.783)	−0.139 (0.743)
		Control + SS‐31	−0.868 (0.005)**	−0.893 (0.003)**	−0.796 (0.018)*
		SD + saline SD + SS‐31	−0.825 (0.012)* −0.833 (0.010)*	−0.761 (0.028)* −0.726 (0.041)*	−0.858 (0.006)** −0.759 (0.029)*
	Percentage of time swam	Control + saline	−0.843 (0.009)**	−0.117 (0.783)	−0.139 (0.743)
		Control + SS‐31	−0.868 (0.005)**	−0.893 (0.003)**	−0.796 (0.018)*
		SD + saline SD + SS‐31	−0.825 (0.012)* −0.833 (0.010)*	−0.761 (0.028)* −0.726 (0.041)*	−0.858 (0.006)** −0.759 (0.029)*

Abbreviations: IL, interleukin; SD, sleep deprivation; TNF, tumor necrosis factor. *Denates a significant correlation (*P < 0.05; **P < 0.01).

**TABLE 3:** Correlation between the performance of Morris water maze test (MWM) and hippocampal mRNA levels [*r* (*p*)].

Tasks	Indexes	Groups	mRNA levels					
			NF‐κb	Sirt1	Pgc1α	Bdnf	Psd‐95	Syn
Morris water maze test	Escape latency	Control + saline	0.540 (0.167)	−0.365 (0.375)	−0.716 (0.046)*	−0.668 (0.070)	−0.753 (0.031)*	−0.756 (0.030)*
		Control + SS‐31	0.746 (0.033)*	−0.709 (0.049)*	−0.833 (0.010)*	−0.706 (0.050)	−0.921 (0.001)**	−0.734 (0.038)*
		SD + saline SD + SS‐31	0.896 (0.003)** 0.903 (0.002)**	−0.869 (0.005)** −0.973 (0.000)**	−0.886 (0.003)** −0.842 (0.009)**	−0.839 (0.009)** −0.788 (0.020)*	−0.930 (0.001)** −0.940 (0.001)**	−0.884 (0.004)** −0.870 (0.005)**
	Distance	Control + saline Control + SS‐31	0.458 (0.254) 0.754 (0.031)*	−0.663 (0.073) −0.716 (0.046)*	−0.754 (0.031)* −0.830 (0.011)*	−0.580 (0.132) −0.712 (0.048)*	−0.705 (0.051) −0.896 (0.003)**	−0.732 (0.039)* −0.759 (0.029)*
		SD + saline	0.862 (0.006)**	−0.872 (0.005)**	−0.799 (0.017)*	−0.815 (0.014)*	−0.911 (0.002)**	−0.881 (0.004)**
		SD + SS‐31	0.885 (0.003)**	−0.966 (0.000)**	−0.822 (0.012)*	−0.764 (0.027)*	−0.951 (0.000)**	−0.826 (0.011)*
	Percentage of distance swam	Control + saline	−0.808 (0.015)*	0.595 (0.120)	0.847 (0.008)**	0.610 (0.108)	0.604 (0.113)	0.840 (0.009)**
		Control + SS‐31	−0.758 (0.029)*	0.718 (0.045)*	0.761 (0.028)*	0.839 (0.009)**	0.884 (0.004)**	0.771 (0.025)*
		SD + saline SD + SS‐31	−0.890 (0.003)** −0.871 (0.005)**	0.723 (0.043)* 0.883 (0.004)**	0.952 (0.000)** 0.818 (0.013)*	0.807 (0.016)* 0.778 (0.023)*	0.890 (0.003)** 0.835 (0.010)**	0.879 (0.004)** 0.812 (0.014)*
	Percentage of time swam	Control + saline	−0.808 (0.015)*	0.595 (0.120)	0.847 (0.008)	0.610 (0.108)	0.604 (0.113)	0.840 (0.009)**
		Control + SS‐31	−0.758 (0.029)*	0.718 (0.045)*	0.761 (0.028)*	0.839 (0.009)**	0.884 (0.004)**	0.771 (0.025)*
		SD + saline SD + SS‐31	−0.890 (0.003)** −0.871 (0.005)**	0.723 (0.043)* 0.883 (0.004)**	0.952 (0.000)** 0.818 (0.013)*	0.807 (0.016)* 0.778 (0.023)*	0.890 (0.003)** 0.835 (0.010)**	0.879 (0.004)** 0.812 (0.014)*

Abbreviations: Bdnf, brain‐derived neurotrophic factor; Nfkb, nuclear factor‐kappa B; Pgc1a, peroxisome proliferator‐activated receptor γ coactivator alpha; Psd‐95, postsynaptic density‐95; SD, sleep deprivation; Sirt1, sirtuin 1; Syn, synaptophysin.*Denates a significant correlation (*P < 0.05; **P < 0.01).

**TABLE 4:** Correlation between the performance of Morris water maze test (MWM) and hippocampal synaptic proteins levels [*r* (*p*)].

Tasks	Indexes	Groups	Proteins levels					
			NF‐κB	Sirt1	PGC‐1α	BDNF	PSD‐95	SYN
Morris water maze test	Escape latency	Control + saline	0.820 (0.046)*	−0.770 (0.073)	−0.658 (0.155)	−0.763 (0.078)	−0.718 (0.108)	−0.746 (0.088)
		Control + SS‐31	0.939 (0.005)**	−0.927 (0.008)**	−0.852 (0.031)*	−0.898 (0.015)*	−0.836 (0.038)*	−0.904 (0.013)*
		SD + saline SD + SS‐31	0.959 (0.002)** 0.853 (0.031)*	−0.894 (0.016)* −0.906 (0.013)	−0.888 (0.018)* −0.847 (0.033)*	−0.994 (0.000)** −0.880 (0.021)*	−0.973 (0.001)** −0.913 (0.011)*	−0.988 (0.000)** −0.958 (0.003)**
	Distance	Control + saline Control + SS‐31	0.394 (0.440) 0.835 (0.039)*	−0.533 (0.276) −0.888 (0.018)*	−0.323 (0.533) −0.907 (0.013)*	−0.358 (0.486) −0.968 (0.002)**	−0.243 (0.643) −0.833 (0.040)*	−0.369 (0.472) −0.977 (0.001)**
		SD + saline	0.860 (0.028)*	−0.873 (0.023)*	−0.699 (0.122)	−0.936 (0.006)**	−0.855 (0.030)*	−0.902 (0.014)*
		SD + SS‐31	0.944 (0.005)**	−0.866 (0.026)*	−0.919 (0.009)**	−0.915 (0.011)*	−0.864 (0.026)*	−0.823 (0.044)*
	Percentage of distance swam	Control + saline	−0.811 (0.050)	0.619 (0.190)	0.453 (0.367)	0.469 (0.349)	0.725 (0.103)	0.559 (0.249)
		Control + SS‐31	−0.899 (0.015)*	0.929 (0.007)**	0.856 (0.030)*	0.869 (0.025)*	0.830 (0.041)*	0.852 (0.031)*
		SD + saline SD + SS‐31	−0.967 (0.002)** −0.966 (0.002)**	0.922 (0.009)** 0.997 (0.000)**	0.971 (0.001)** 0.894 (0.016)*	0.866 (0.026)* 0.986 (0.000)**	0.949 (0.004)** 0.952 (0.003)**	0.910 (0.012)* 0.974 (0.001)**
	Percentage of time swam	Control + saline	−0.811 (0.050)	0.619 (0.190)	0.453 (0.367)	0.469 (0.349)	0.725 (0.103)	0.559 (0.249)
		Control + SS‐31	−0.899 (0.015)*	0.929 (0.007)**	0.856 (0.030)*	0.869 (0.025)*	0.830 (0.041)*	0.852 (0.031)*
		SD + saline SD + SS‐31	−0.967 (0.002)** −0.966 (0.002)**	0.922 (0.009)** 0.997 (0.000)**	0.971 (0.001)** 0.894 (0.016)*	0.866 (0.026)* 0.986 (0.000)**	0.949 (0.004)** 0.952 (0.003)**	0.910 (0.012)* 0.974 (0.001)**

Abbreviations: BDNF, brain‐derived neurotrophic factor; NF‐kB, nuclear factor‐kappa B; PGC‐1α, peroxisome proliferator‐activated receptor γ coactivator alpha; PSD‐95, postsynaptic density‐95; SD, sleep deprivation; Sirt1, sirtuin 1; SYN, synaptophysin.*Denates a significant correlation (*P < 0.05; **P < 0.01).

## 4 DISCUSSION

In the present study, we observed that CSD‐induced alterations in learning and memory function, mitochondrial function, SIRT1 expression, the inflammatory response, and synaptic function could be restored by SS‐31 treatment. Therefore, SS‐31 could be a potential agent in the treatment of cognitive impairment induced by CSD.

### 4.1 SS‐31 improves learning and memory impairment induced by CSD

We assessed cognitive impairment using the novel object recognition and Morris water maze tests in this study, as this is proven to be reliable tests that are strongly related with hippocampal‐dependent learning and memory (Zhang, Chen, et al., 2022). Previous studies showed that sleep dysfunction could affect hormone levels, neural circuits, dendrite density, and synaptic transmission, leading to cognitive decline (Brüning et al., 2019; Voldsbekk et al., 2022). Male Wistar rats subjected to sleep deprivation for 3 days showed spatial memory decline during the Morris water maze test (Duan et al., 2016). Short‐term sleep deprivation of 6 h significantly impaired the memory consolidation in male C57BL/6J mice during the object‐location memory paradigm (Raven et al., 2020). Consistently, we found that CSD for 21 days decreased the NOI during the NOI, increased escape latency and distance, and increased time and distance percent in the SD + SS‐31 group compared to the SD + saline group during the Morris water maze, indicating impaired learning and memory in CSD mice.

The cognitive protective effects of SS‐31 have been extensively studied in various pathological models. One study showed that SS‐31 pretreatment improved isoflurane‐induced cognitive decline through improving mitochondrial function (Wu, Li, et al., 2015). Another study showed that SS‐31 increased the downregulation in the percentage freezing time caused by sepsis‐associated encephalopathy during contextual fear conditioning (Wu, Zhang, et al., 2015). We found that treatment with SS‐31 improved CSD‐induced learning and memory impairment as observed in the novel object recognition and Morris water maze tests. Additionally, the mice from the Control + SS‐31 group showed no significant difference from those in the Control + saline group in the novel object recognition and Morris water maze tests. This suggests that SS‐31 might only have an effect on damaged mitochondria and not on normal mitochondria.

### 4.2 SS‐31 improves mitochondrial dysfunction induced by CSD

Accumulating evidence suggests that integrated mitochondrial function is closely related to cognitive function (Zhuang et al., 2021). The structure and number of mitochondria are not static, as mitochondria grow and divide continuously in a process known as mitochondrial biogenesis to respond to changing energy demands and stressful events. PGC‐1α is an important transcription factor involved in mitochondrial biogenesis, which helps to maintain mitochondrial function by regulating mitochondrial dynamics and energy (Chen et al., 2011; Zhu et al., 2010). PGC‐1α is transferred to the nucleus where it co‐activates nuclear respiratory factor (NRF) and mitochondrial transcription factor A (TFAM). The combination of NRF and TFAM then promotes the replication and expression of mitochondrial genes (Finck & Kelly, 2006; Scarpulla, 2008). Furthermore, SIRT1 is a pivotal factor in the regulation of mitochondrial metabolism, which activates PGC‐1α through histone deacetylation. Isoflurane‐induced anesthesia decreased the expression of SIRT1 and PGC‐1α and impaired cognitive function in aging rats (Yang et al., 2020). Triptolide activated the SIRT1/PGC‐1α signaling pathway to improve cognitive impairment in rats with vascular dementia (Yao et al., 2019). In the current study, we found that the mRNA and protein levels of SIRT1 and PGC‐1α were decreased in the SD + saline group after CSD, implying that mitochondrial biogenesis dysfunction was involved in the cognitive impairment induced by CSD. Moreover, SS‐31 increased the levels of SIRT1 and PGC‐1α under the sleep deprivation condition, suggesting that SS‐31 improves CSD‐induced cognitive and mitochondrial dysfunction by modulating the SIRT1/PGC‐1α signaling pathway.

### 4.3 SS‐31 improves inflammatory response induced by CSD

Progressive neuroinflammation plays a primary role in the cognitive impairment related with neurodegenerative diseases such as Alzheimer's disease and Huntington's disease (Bains et al., 2022; Decandia et al., 2023). Neuroinflammation influences cell proliferation and differentiation, synaptic plasticity, and cognitive function (Bai et al., 2023; Cangalaya et al., 2023). The levels of pro‐inflammatory cytokines were found to be increased in the peripheral blood of patients with sleep disorders (Irwin et al., 2016; Xia et al., 2021). In addition, sleep deprivation significantly elevated the levels of proinflammatory cytokines including IL‐1β, IL‐6, and TNF‐α in the hippocampus and resulted in cognitive impairment during the novel object recognition test (Lu et al., 2022). In line with these previous studies, the results showed that the levels of IL‐1β, IL‐6, and TNF‐α were increased in the hippocampus of the mice exposed to CSD, which contributed to the observed cognitive decline.

Growing evidence suggests that mitochondria are directly linked to the immune system and inflammatory response. NF‐κB and NOD‐like receptor thermal protein domain associated protein 3 (NLPR3) are important transcription factors associated with the inflammatory response. The ROS produced by mitochondria activate NF‐κB and NLPR3, which in turn promotes the excessive release of the pro‐inflammatory cytokines (Nakahira et al., 2011; Sato et al., 2004; Zhou et al., 2011). Importantly, a previous study showed that treatment with SS‐31 significantly ameliorated isoflurane‐induced cognitive deficits and inflammatory responses by decreasing the levels of NF‐κB, NLPR3, and ROS (Wu et al., 2015). Our results showed that SS‐31 reduced the elevated levels of NF‐κB and proinflammatory cytokines in the SD + SS‐31 group, suggesting that SS‐31 could suppress the CSD‐induced inflammatory response by improving mitochondrial dysfunction.

### 4.4 SS‐31 improves synaptic dysfunction induced by chronic sleep deprivation

BDNF is mainly expressed in the hippocampus, where it controls neuronal maturation and survival (Mosiołek et al., 2022). A large body of evidence suggests that BDNF plays an important role in synaptic plasticity and synaptic transmission that is considered to underlie learning and memory (Liu et al., 2022; Wang et al., 2023). SYN and PSD‐95 are synaptic proteins that are closely related to synaptic maturation, transmission, and remodeling (Aguiar et al., 2022; Reinés et al., 2008). Decreased expression levels of BDNF, SYN, and PSD‐95 have been associated with the pathogenesis of cognitive dysfunction (Wang & Zhao, 2016; Hong et al., 2020). The present study showed that CSD decreased the mRNA and protein levels of BDNF, SYN, and PSD‐95 in mice, which contributed to cognitive deficits induced by CSD. Furthermore, pretreatment with SS‐31 attenuated the reduction of BDNF, SYN, and PSD‐95 induced by CSD, which is consistent with a previous study showing that SS‐31 prevented the lipopolysaccharide‐induced downregulated of SYN and PSD‐95 through modulating the BDNF signaling pathway (Zhao et al., 2019).

### 4.5 Correlation between the cognition‐related performance and the markers of mitochondrial biogenesis, proinflammatory response, and synaptic function

Emerging evidence also suggests that mitochondrial biogenesis function is closely related to cognitive function. Our previous study showed that the decreased expression level of PGC‐1α was correlated with poor cognitive performance induced by prenatal inflammatory exposure during the Morris water maze test (Zhuang et al., 2021). In the current study, the downregulated expression of PGC‐1α was also considered to play a role in the learning and memory impairment induced by CSD, as reflected by the correlation between the expression level of PGC‐1α and indicators of the Morris water maze test in the SD + SD group. Clinical studies have found that the levels of anti‐inflammatory cytokines are positively correlated with cognitive performance associated with sleep disorders (He et al., 2021). Thus, our preclinical study confirms that the levels of pro‐inflammatory cytokines are correlated with the cognitive impairment induced by CSD. Additionally, the levels of synaptic plasticity‐associated proteins reflect the function of synaptic connection, synaptic transmission, and synaptic plasticity and are closely associated with cognitive function (Sadigh‐Eteghad et al., 2020). We also found that the decreased expression levels of BDNF, SYN, and PSD‐95 were correlated with CSD‐induced cognitive impairment.

Our study has some limitations. First, we have not further evaluated mitochondrial function and oxidative stress in the hippocampus, including the mitochondrial electron transport system, ROS, and antioxidant defense enzymes. Second, we determined the mitochondrial biogenesis in the whole hippocampus; thus, it was not possible to distinguish whether mitochondrial dysfunction in the neurons or glial cells was responsible for the inflammation and synaptic dysfunction induced by CSD. Third, we only selected male mice for behavioral experiment and did not evaluate the sex effects of CSD on cognitive function even though previous studies have reported that women are more susceptible to develop sleep disorders than men. Finally, the markers of mitochondrial function, inflammation, and synaptic function were only examined in the hippocampus and were not examined in other regions associated with cognitive function.

## 5 CONCLUSION

In conclusion, our results demonstrated that inflammation and synaptic dysfunction induced by mitochondrial biogenesis dysfunction are involved in the cognitive impairment caused by CSD. The mitochondrial‐targeted antioxidant SS‐31 could improve the CSD‐induced inflammation response, synaptic dysfunction, and cognitive deficits by attenuating mitochondrial biogenesis dysfunction. This work suggests that SS‐31 could be a novel agent for patients with chronic insomnia‐associated cognitive impairment. Future studies are needed to explore the protective effects of SS‐31 on mitochondrial dysfunction and cognitive deficits in more pathological models.

## AUTHOR CONTRIBUTIONS

**Yue‐Ming Zhang**: Conceptualization; investigation; methodology; software; supervision; writing—original draft. **Ya‐Tao Wang**: Conceptualization; investigation; validation; methodology; software; formal analysis; writing—original draft. **Ru‐Meng Wei**: Conceptualization; investigation; validation; visualization; formal analysis; supervision; writing—original draft. **Xue‐Yan Li**: Data curation; supervision; formal analysis; validation; funding acquisition; conceptualization; investigation. **Bao‐Ling Luo**: Conceptualization; investigation; funding acquisition; visualization; validation; software; supervision; resources. **Jing‐Ya Zhang**: Conceptualization; writing—original draft; funding acquisition; validation; methodology; software; supervision; data curation. **Kai‐Xuan Zhang**: Conceptualization; investigation; validation; methodology; formal analysis; software; supervision; resources. **Shi‐Kun Fang**: Conceptualization; investigation; writing—original draft; validation; methodology; software; formal analysis; supervision; data curation. **Xue‐Chun Liu**: Conceptualization; funding acquisition; validation; methodology; software; formal analysis; data curation; supervision; writing—review and editing. **Gui‐Hai Chen**: Conceptualization; investigation; funding acquisition; methodology; validation; visualization; software; supervision; data curation; resources; writing—review and editing.

## CONFLICT OF INTEREST STATEMENT

The authors report no conflicts of interest in this work.

### PEER REVIEW

The peer review history for this article is available at https://publons.com/publon/10.1002/brb3.3508.

## Supporting information

Supporting Information

Supporting Information

## Data Availability

The data that support the findings of this study are available from the corresponding author upon reasonable request.
