# Supplementary material for: Mitochondrial antioxidant elamipretide improves learning and memory impairment induced by chronic sleep deprivation in mice
Source: Brain Behav. 2024 Apr 30;14(5):e3508. doi: 10.1002/brb3.3508 (PMC11061203; doi:10.1002/brb3.3508)
Supplement: Supplementary file 1 — Supporting Information [file BRB3-14-e3508-s002.docx]

**Table 1.** The time and distance percentage of the four quadrants on the first day of the Morris water maze test

| Time percent (%) | Control+saline | Control+SS-31 | SD+saline | SD+SS-31 | Treatment [ *F*(*p*)] | Drug [ *F*(*p*)] | Interaction [ *F*(*p*)] |
| --- | --- | --- | --- | --- | --- | --- | --- |
| First quadrant | 26.13 ± 1.23 | 22.50 ± 1.71 | 24.25 ± 1.88 | 25.63 ± 1.88 | 0.14 | 0.44 | 0.15 |
| Second quadrant | 24.25 ± 1.35 | 24.63 ± 2.12 | 26.50 ± 1.13 | 25.75 ± 1.22 | 1.25 | 0.02 | 0.71 |
| Third quadrant | 25.88 ± 1.74 | 25.13 ± 1.20 | 23.13 ± 1.81 | 23.75 ± 1.80 | 1.55 | 0.00 | 0.68 |
| Forth quadrant | 23.75 ± 1.72 | 27.75 ± 1.39 | 26.13 ± 1.26 | 24.88 ± 1.71 | 0.03 | 0.81 | 2.94 |
| Distance percent (%) |  |  |  |  |  |  |  |
| First quadrant | 26.38 ± 1.22 | 23.00 ± 1.64 | 24.50 ± 1.89 | 25.88 ± 2.00 | 0.09 | 0.34 | 1.92 |
| Second quadrant | 24.63 ± 1.36 | 24.38 ± 2.05 | 26.38 ± 1.34 | 26.00 ± 1.36 | 1.17 | 0.04 | 0.00 |
| Third quadrant | 25.75 ± 1.63 | 25.00 ± 1.15 | 23.25 ± 1.70 | 23.75 ± 1.79 | 1.40 | 0.01 | 0.16 |
| Forth quadrant | 23.50 ± 1.66 | 27.63 ± 1.44 | 26.13 ± 1.36 | 24.38 ± 1.52 | 0.04 | 0.63 | 3.85 |

Abbreviations: SD, sleep deprivation.

**Table 2.** The time and distance percentage of the four quadrants on the memory phase of the Morris water maze test

| Time percent (%) | Control+saline | Control+SS-31 | SD+saline | SD+SS-31 | Treatment [ *F*(*p*)] | Drug [ *F*(*p*)] | Interaction [ *F*(*p*)] |
| --- | --- | --- | --- | --- | --- | --- | --- |
| First quadrant | 19.75 ± 0.96 | 19.38 ± 1.19 | 25.38 ± 1.67 | 19.63 ± 1.09 | 5.47* | 5.95* | 4.58* |
| Second quadrant | 19.13 ± 0.90 | 20.38 ± 0.86 | 24.13 ± 1.59 | 20.25 ± 1.11 | 4.48* | 1.30 | 4.95* |
| Third quadrant | 20.88 ± 1.27 | 18.75 ± 0.96 | 24.50 ± 1.21 | 22.75 ± 1.63 | 8.71* | 2.25 | 0.02 |
| Forth quadrant | 40.25 ± 2.14 | 42.00 ± 2.04 | 26.00 ± 2.07 | 37.38 ± 2.38 | 19.12** | 9.25** | 4.97* |
| Distance percent (%) |  |  |  |  |  |  |  |
| First quadrant | 19.38 ± 1.00 | 19.13 ± 1.14 | 25.50 ± 1.68 | 19.50 ± 0.94 | 7.03* | 6.50* | 5.50* |
| Second quadrant | 20.00 ± 0.85 | 20.75 ± 0.77 | 24.25 ± 1.52 | 20.25 ± 1.10 | 2.91 | 2.19 | 4.67* |
| Third quadrant | 20.38 ± 1.22 | 18.63 ± 0.94 | 24.63 ± 1.18 | 21.88 ± 1.58 | 8.99* | 3.24 | 0.16 |
| Forth quadrant | 40.25 ± 2.14 | 42.00 ± 2.04 | 25.00 ± 2.07 | 38.38 ± 2.36 | 19.12** | 12.28** | 7.25* |

*Denotes significant correlation (^*^*P* < 0.05; ^**^*P* < 0.01)

Abbreviations: SD, sleep deprivation.
