# Supplementary figures and images for: Mitochondrial antioxidant elamipretide improves learning and memory impairment induced by chronic sleep deprivation in mice
Source: Brain Behav. 2024 Apr 30;14(5):e3508. doi: 10.1002/brb3.3508 (PMC11061203; doi:10.1002/brb3.3508)

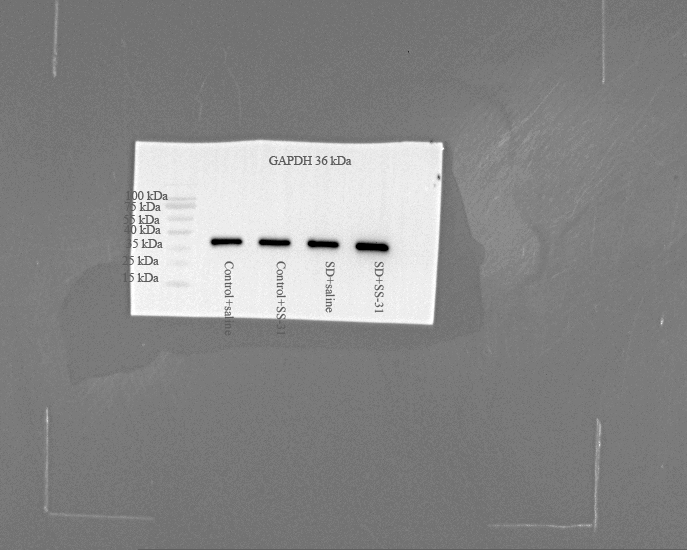

Supplement: Supplementary file 2 — Supporting Information [file BRB3-14-e3508-s001.zip › Supplementary figure/Supplementary figure 1-GAPDH.tif]

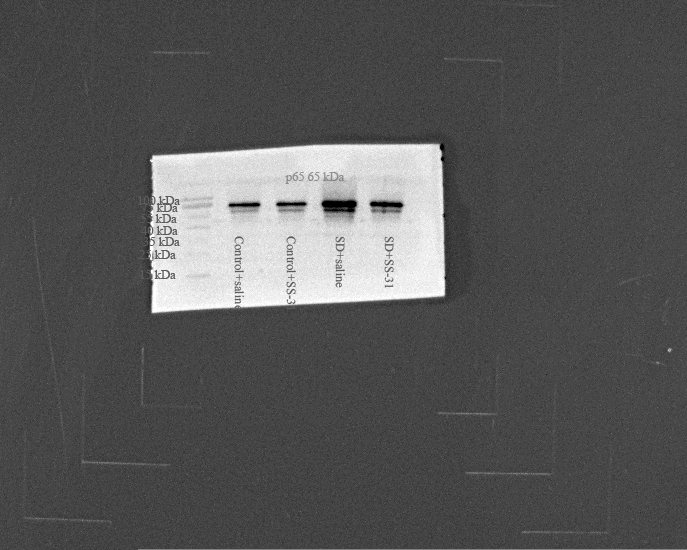

Supplement: Supplementary file 2 — Supporting Information [file BRB3-14-e3508-s001.zip › Supplementary figure/Supplementary figure 1-NF-kB.tif]

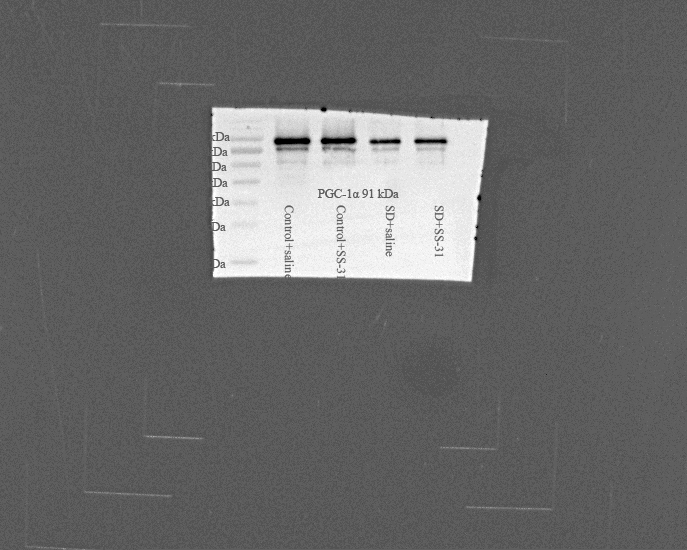

Supplement: Supplementary file 2 — Supporting Information [file BRB3-14-e3508-s001.zip › Supplementary figure/Supplementary figure 1-PGC-1α.tif]

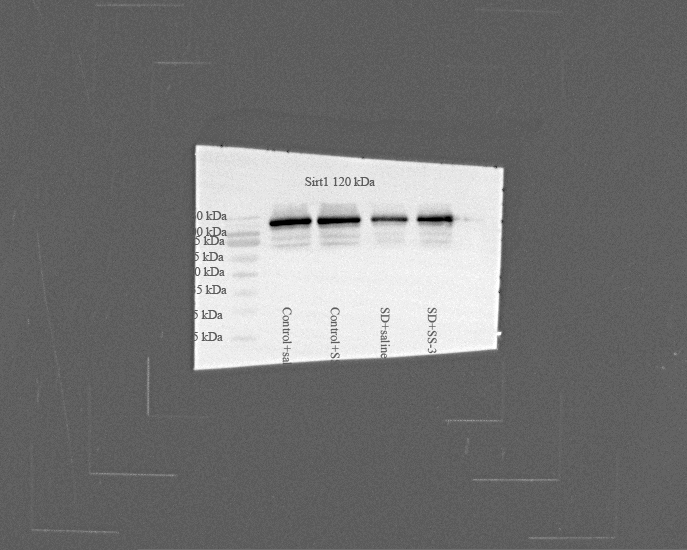

Supplement: Supplementary file 2 — Supporting Information [file BRB3-14-e3508-s001.zip › Supplementary figure/Supplementary figure 1-sirt1.tif]

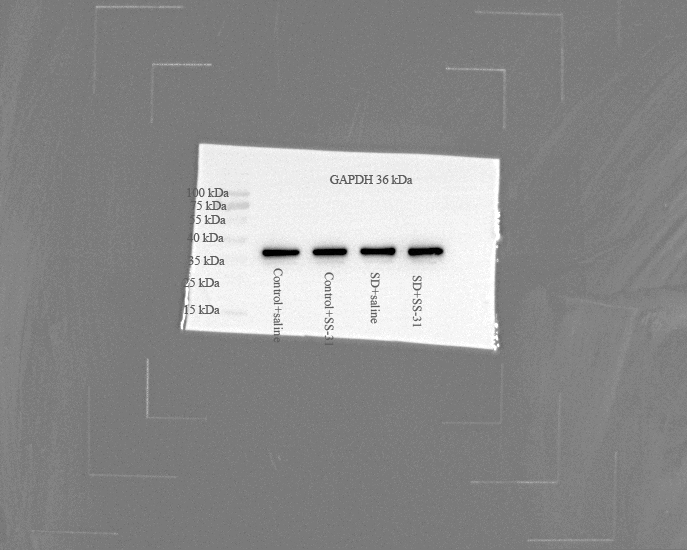

Supplement: Supplementary file 2 — Supporting Information [file BRB3-14-e3508-s001.zip › Supplementary figure/Supplementary figure 2-GAPDH.tif]

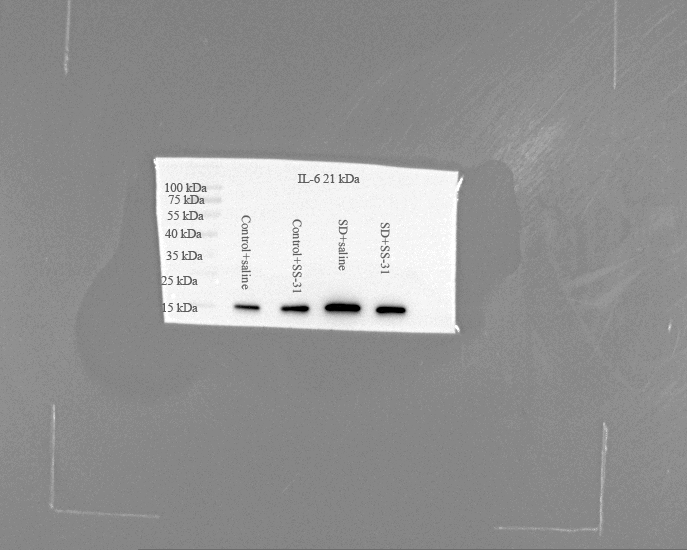

Supplement: Supplementary file 2 — Supporting Information [file BRB3-14-e3508-s001.zip › Supplementary figure/Supplementary figure 2-IL-6.tif]

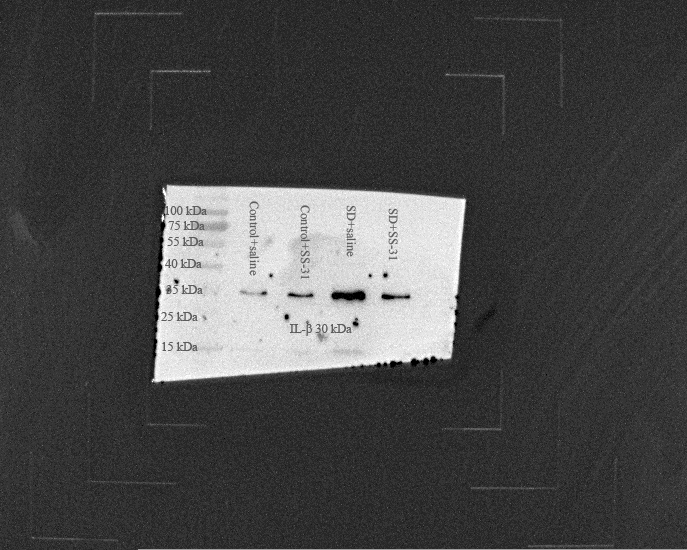

Supplement: Supplementary file 2 — Supporting Information [file BRB3-14-e3508-s001.zip › Supplementary figure/Supplementary figure 2-IL-β.tif]

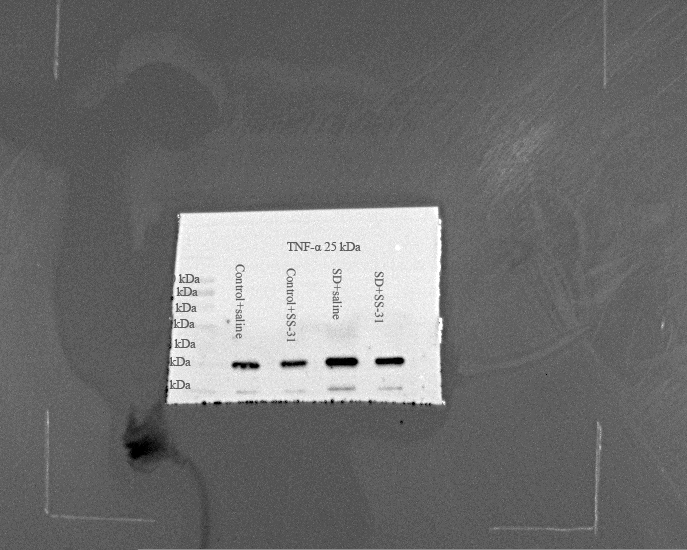

Supplement: Supplementary file 2 — Supporting Information [file BRB3-14-e3508-s001.zip › Supplementary figure/Supplementary figure 2-TNF-α.tif]

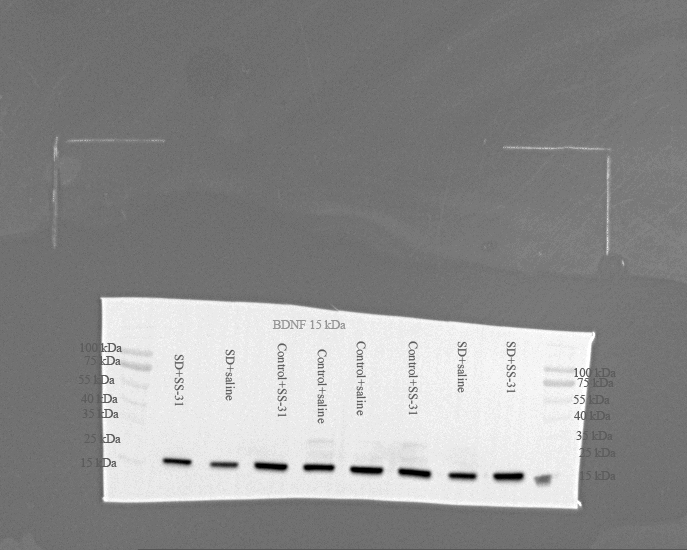

Supplement: Supplementary file 2 — Supporting Information [file BRB3-14-e3508-s001.zip › Supplementary figure/Supplementary figure 3-BDNF.tif]

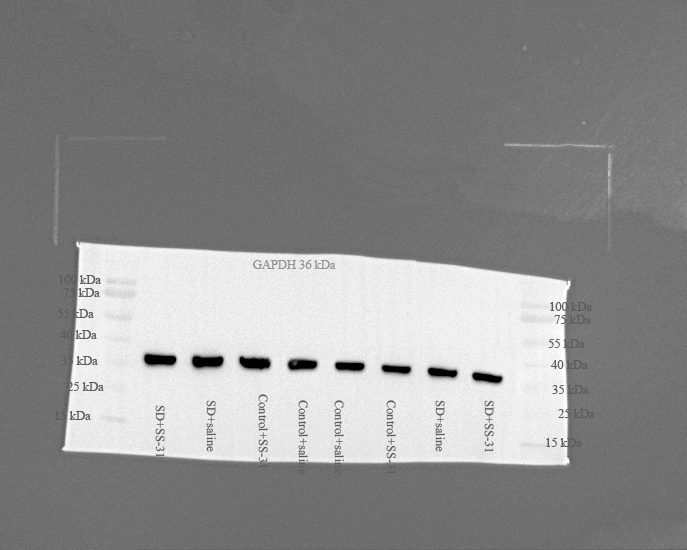

Supplement: Supplementary file 2 — Supporting Information [file BRB3-14-e3508-s001.zip › Supplementary figure/Supplementary figure 3-GAPDH.tif]

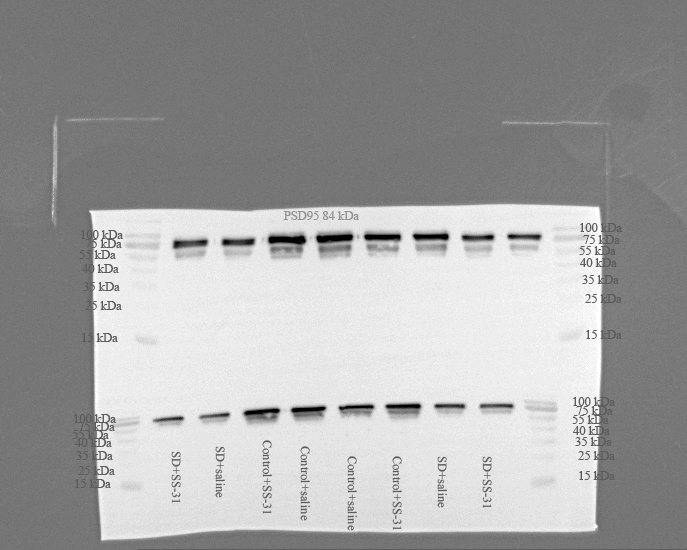

Supplement: Supplementary file 2 — Supporting Information [file BRB3-14-e3508-s001.zip › Supplementary figure/Supplementary figure 3-PSD-95.tif]

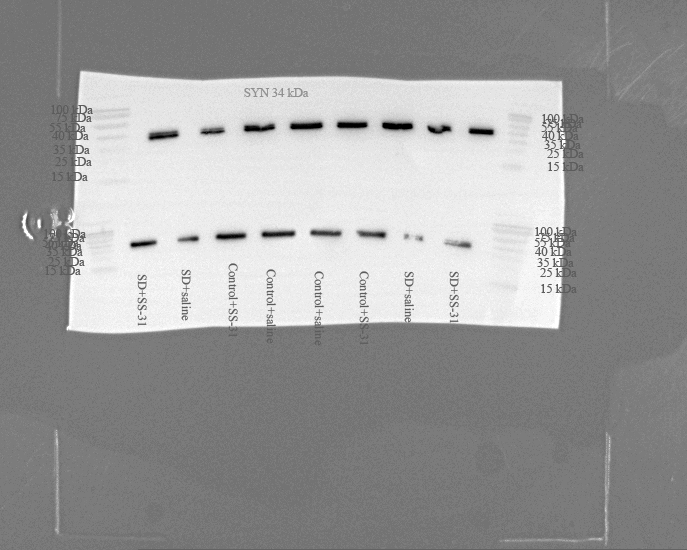

Supplement: Supplementary file 2 — Supporting Information [file BRB3-14-e3508-s001.zip › Supplementary figure/Supplementary figure 3-SYN.tif]
